# Wnt family members regulating osteogenesis and their origins

**DOI:** 10.1007/s00774-024-01554-y

**Published:** 2024-09-16

**Authors:** Yasuhiro Kobayashi, Rina Iwamoto, Zhifeng He, Nobuyuki Udagawa

**Affiliations:** 1 Department of Hard Tissue Research, Institute for Oral Science, Matsumoto Dental University, 1780 Hirooka Gohara, Shiojiri, Nahano 399-0781 Japan; 2 Department of Biochemistry, Matsumoto Dental University, 1780 Hirooka Gohara, Shiojiri, Nagano 399-0781, Japan

**Keywords:** Wnt, Osteoblast, Bone formation, Wnt signals

## Abstract

Wnt signaling plays an important role in the regulation of bone metabolism. Wnt activates the β-catenin-mediated canonical pathway and β-catenin-independent non-canonical pathway. When Wnt ligands bind to the co-receptors low density lipoprotein receptor-related protein (Lrp)5 or Lrp6, and a seven-transmembrane receptor frizzled, the canonical pathway is activated. On the other hand, when Wnt ligands bind to the receptor complex consisting of the co-receptor receptor tyrosine kinase-like orphan receptor (Ror)1 and Ror2 or Ryk and frizzled, the non-canonical pathway is activated. An analysis of loss-of-function and gain-of-function mutations in molecules involved in Wnt signaling (ligands, receptors, and inhibitors) has revealed the mechanisms by which Wnt signaling regulates bone metabolism. In this review, based on transcriptome analyses of Wnt expression in bone tissues including single cell RNA sequence analysis and previous literatures, we herein introduce and discussed the latest findings on the mechanisms by which Wnt ligand mutations impair bone metabolism, especially bone formation.

## Introduction

**Fig. 1:**
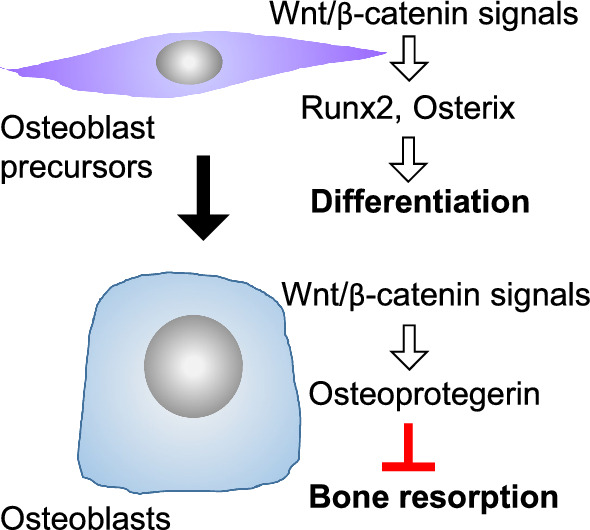
Wnt/β-catenin signals in osteoblast precursors induce the expression of Runx2 and Osterix, thereby promoting osteoblastogenesis. Wnt/β-catenin signals in mature osteoblasts induce the expression of osteoprotegerin and then inhibit bone resorption

Wnt signals regulate bone metabolism. Wnt ligands activate β-catenin-dependent canonical and -independent non-canonical pathways [1, 2]. Wnt ligands bind to the receptor complex composed of frizzled and low-density lipoprotein receptor-related protein (Lrp) 5 or Lrp6, and then activate Wnt/β-catenin signals. In osteoblast precursors, the activation of Wnt/β-catenin signals induces the expression of master transcription factors for osteoblastogenesis such as Runx2 and Osterix, and the differentiation of osteoblasts is then initiated [3, 4] (Fig. 1). In addition, the activation of Wnt/β-catenin signals in mature osteoblasts induces the expression of osteoprotegerin (encoding Tnfrsf11b, Opg), a decoy receptor of RANKL, and then suppresses osteoclast differentiation, thereby inhibiting bone resorption [5]. Therefore, Wnt/β-catenin signals increase bone mass by activating bone formation and inhibiting bone resorption. Wnt signals are suppressed by endogenous antagonists, such as sclerostin [6, 7], dickkopf family members, and secreted frizzled-related protein (Sfrp) family members [8].

Wnt5a, a typical non-canonical Wnt ligand, binds to co-receptors Ror1, Ror2, and Ryk. This ligand-receptor complex activates non-canonical Wnt pathways, such as the planner cell polarity (PCP) pathway and Wnt/Ca^2+^ pathway [2]. Wnt5a binds to the Ror2 receptor in osteoclast precursors and activates JNK-Sp1 signals, which, in turn, enhance the expression of Rank. Therefore, Wnt5a promotes the Rankl-induced formation of osteoclasts [9]. Moreover, Wnt5a-Ror2 signals have been shown to positively regulate the bone resorbing activity of osteoclasts [10, 11]. Wnt5a regulates the small G protein Rho, its effector PKN3, and c-Src to govern bone-resorbing activity.

Loss-of-function mutations and gain-of-function mutations in the molecules involved in Wnt signals such as Lrp5, β-catenin, and a Wnt inhibitor sclerostin reveal the mechanisms by Wnt signals regulate bone metabolism. Since there are 19 Wnt ligands in humans and mice [12], difficulties are associated with clarifying their roles in bone metabolism using the present gene knockout strategy. Nineteen Wnt ligands form a sophisticated system that strictly regulates bone metabolism. However, the Wnt ligands that are critical for bone metabolisms and the type of cells that express Wnt ligands remain unclear. Therefore, this review summarizes information on the expression data of Wnt ligands in bone tissues and the bone phenotypes of Wnt ligand mutations and discusses Wnt ligands that are important for bone mass.

### Sources of Wnt ligands for increasing bone mass

Wntless (also known as Evi and GPR177) is an essential molecule for transporting Wnt ligands from the endoplasmic reticulum to the plasma membrane [13]. Cells lacking the Wntless gene cannot secrete all Wnt ligands. To identify cell types that secrete Wnt ligands and regulate bone mass, the bone phenotype of Wntless conditional knockout (cKO) mice was analyzed [14, 15]. Osteocalcin-Cre; Wntless (Wls)^fl/fl^ mice showed severe bone loss accompanied by osteogenesis imperfecta (OI) [14]. Col(I)-Cre; Wls ^fl/fl^ mice had a similar bone phenotype [15]. Thus, osteoblast lineage cell-specific Wls cKO mice exhibited a severe low bone mass phenotype (approximately 80% and 90% decrease in Bone Volume /Tissue Volume of Osteocalcin-Cre; Wntless (Wls)^fl/fl^ mice and that of Col (I)-Cre; Wls ^fl/fl^ mice, respectively). These findings suggest that Wnt ligands derived from osteoblast lineage cells, including osteoblasts and osteocytes, but not other types of cells, are crucially important for maintaining bone mass. However, the Wnt ligands expressed in each differentiation stage of osteoblast cell lineages remain unclear. Therefore, several single-cell RNA sequence (scRNA seq) datasets were re-analyzed and summarized together with the previous findings using real-time PCR and ISH analysis in Table 1. Osteoblast-lineage cells express Wnt ligands in the following order: Wnt4 > Wnt5a > Wnt5b > Wnt16 > Wnt11 > Wnt10b > Wnt10a = Wnt2 = Wnt1.

**Table 1:** Summary of Wnt expressions in bone tissues

	Calvarial cells	Cortical bone	Cortical Obs	Calvarial Obs	Calvarial cells	Obs	BMS	Obs	BMS	Chondrocyte	Obs	BMS	Chondrocyte
Wnt1		+ +	+	+		+	+	+		+	+		
Wnt2	+	+			+ +			+			+ +		
Wnt2b	+	+					+	+		+			+
Wnt3		+											
Wnt3a		+	+										
Wnt4	+ +	+ +	7 +	+	+ +	4 +	5 +	4 +	5 +	+ + +	5 +	10 +	5 +
Wnt5a	8 +	5 +	+ + +	7 +	+ +	+ +	+ +	+ + +	+ +	+ +	4 +	4 +	4 +
Wnt5b	+ +	5 +	+ + +	+ + +		+ +	+ +	+ + +		+ +	4 +	6 +	8 +
Wnt6		+						+	+		+ + +	+ + +	
Wnt7a		+											
Wnt7b		+	10 +	+ +			+	+ +		+ +			
Wnt8a		+								+			
Wnt8b		+								+			
Wnt9a		+ +						+ +					
Wnt9b	+	+	+			+	+						
Wnt10a		+			4 +	+ +					+ +		
Wnt10b		5 +	+ + +	+ + +				+ +		+		+ +	+ +
Wnt11	+ +	+	+ + +	+ + +	+ +	+		+	+ +	+ +	+ + +	+ + +	4 +
Wnt16	+ + +	+ +	7 +	+ + +	+ +		+ +	+ + +			+ + +		
Reference DOI	https://doi.org/10.1038/nm.2653	https://doi.org/10.1073/pnas1420463111	https://doi.org/10.1016/j.bone.201212.052	https://doi.org/10.1016/j.bone.201212.052	https://doi.org/10.1073/pnas1120407109	https://doi.org/10.3389/fendo.2023.1063083	https://doi.org/10.3389/fendo.2023.1063083	https://doi.org/10.7554/eLife.54695	https://doi.org/10.7554/eLife.54695	https://doi.org/10.7554/eLife.54695	https://doi.org/10.1016/j.cell.201904.040	https://doi.org/10.1016/j.cell.201904.040	https://doi.org/10.1016/j.cell.201904.040
GEO Accession						GSE208152	GSE208152	GSE145477	GSE145477	GSE145477	GSE128423	GSE128423	GSE128423
Methods	RT-PCR	ISH	RT-PCR	RT-PCR	semi-quantitative PCR	scRNA seq	scRNA seq	scRNA seq	scRNA seq	scRNA seq	scRNA seq	scRNA seq	scRNA seq

*Obs* Osteoblasts, *BMS* Bone marrow stromal cells, *ISH* In situ hybridation, *scRNA seq* single cell RNA sequence

### Wnt1

The expression of Wnt1 in bone tissues was lower than that of other Wnt ligands, such as Wnt4 and Wnt5a (Table 1); however, many studies demonstrated that the involvement of loss-of-function mutations in Wnt1 in the low bone mass phenotype in humans and mice, as described below. The discrepancy between the expression of Wnt1 and the bone phenotype in patients and mice with Wnt1 mutations may be attributed to the following factors; (1) the expression of Wnt1 mRNA was higher in fetuses and childhood than in adulthood, because the majority of transcriptome data was obtained from adult tissues (Table 1), and (2) difficulties are associated with isolating intact osteocytes that highly express Wnt1 for RT-PCR and scRNA seq analysis.

In 2013, WNT1 was identified as the causative gene of the early-onset osteoporosis (EOOP) and osteogenesis imperfect (OI) in humans [16–19]. Wnt1 is expressed in the femurs, spleen, and brain. B220 + cells in bone marrow highly expressed Wnt1 mRNA. Furthermore, a lineage-tracing study using Wnt1-Cre; Rosa26 mT/mG mice showed that Wnt1 is highly expressed in osteocytic cells in the subchondral bone, but was expressed at lower levels in osteocytic cells in cortical bone. Therefore, Wnt1-derived from non-osteoblast lineage cells has been suggested to affect bone mass. Joeng et al. generated osteocyte-specific Wnt1 cKO mice (Dmp-1Cre; Wnt1^fl/fl^) and examined their bone phenotypes [20]. These Wnt1 cKO mice showed spontaneous bone fractures at a rate of 67%. Marked reduction were observed in trabecular bone mass and cortical thickness in these mice with defects in osteoblast functions, whereas the number of osteoblasts and osteoclasts remained unchanged. Furthermore, increases in trabecular bone volume and cortical thickness were reported in osteocyte-specific Wnt1-overexpressing mice (Dmp1-Cre; Rosa26Wnt1^/ +^) and were associated with the elevated bone formation markers, such as the number of osteoblasts and the bone formation rate [20]. Wnt1 enhanced the expression of osteoblast marker genes, such as Runx2 and Alpl through the activation of mTORC1 signals. The treatment of the Wnt1-related osteogenesis imperfecta mouse model Wnt1^sw/sw^ with anti-sclerostin antibodies did not completely rescue their osteoporotic phenotype. These findings indicate that osteocyte-derived Wnt1 promotes bone accrual during bone growing periods and is important for bone anabolic effects of the anti-sclerostin antibodies.

The role of Wnt1 derived from bone marrow stromal cells was also investigated [21]. Limb mesenchymal cell-specific Wnt1 cKO mice (Prrx1-Cre; Wnt1^fl/fl^) mice showed that a similar bone phenotype to osteocyte-specific Wnt1 cKO mice, suggesting that Wnt1 produced locally, but not in the circulation, is critical for bone mass, and also that stromal cell-derived Wnt1 also contributes to bone mass in additions to osteoblast-linage cell-derived Wnt1. It is important to note that the expression of cre-recombinase was detected in limb osteoblasts and osteocytes as well as limb mesenchymal cells because some mesenchymal cells differentiate into osteoblasts and osteocytes [22].

WNT1 mutations in EOOP and OI XV patients, were investigated using mouse models. Mouse carrying mutations (G177C) that cause OI XV had a markedly reduced bone mass, a decreased number of osteoblasts, and a lower bone formation rate [23]. A heterozygous Wnt1 mutation (p.R235W) has been identified in early-onset osteoporosis patients [16]. Wild type WNT1 binds to LRP5 and activates WNT-regulated β-catenin signaling. In contrast, the mutated WNT1 (R235W) showed a significant reduction in these signals, suggesting that WNT1 activates Wnt/β-catenin signals, which, in turn, enhance bone formation. To confirm the bone phenotypes of mice carrying the Wnt1 R235W mutation, the knock-in mice were generated and analyzed [24]. Homozygous mutant mice exhibited a significant reduction of bone volume / tissue volume (BV/TV) and cortical thickness. Furthermore, the intermittent injections of PTH in heterozygous Wnt1 mutant mice induced bone formation to a similar extent to that in WT mice [24]. These findings suggest that the administration of PTH effectively increased bone mass in EOOP patients.

### Wnt4

Wnt4 is highly expressed in osteoblast-lineage cells (Table 1). A genome-wide association study revealed that the Wnt4 gene was related to bone mineral density (BMD). Gregson et al. [25] identified relationships that exceeded genome-wide significance between BMD and four loci: two established BMD-associated loci (MEF2C and WNT4) and two novel loci; (NPR3 associated with lumber spine BMD and SPON1 with total hip BMD). Medina-Gomez et al. [26]. detected variants with pleiotropic effects in eight loci, including seven established BMD loci: WNT4, GALNT3, MEPE, CPED1/WNT16, TNFSF11, RIN3, and PPP6R3/LRP5. GWAS identified three loci (rs30862651 on Chr.1, rs27559568 on Chr.4, and rs33071523 on Chr.17) for osteoblast activity. The locus on Chr. 4 was in an intergenic region between *Wnt4* and Zinc Finger and BTB domain Containing 40 (Zbtb40, a telomere associated protein), homologous to a locus for BMD in humans. Therefore, the bone phenotypes of Wnt4-deficient and Zbtb40-deficient mice were investigated [27]. Since *Wnt4* global knockout mice reportedly die within 24 h of birth due to disrupted kidney function [28], *Prrx1*-Cre; *Wnt4*^*fl/fl*^ mice were generated, and their bone phenotypes were investigated [27]. These mice were expected to exhibits the low bone mass phenotype. Bone parameters were mostly unaffected in cKO mice at maturity; however, reductions were observed in the number of trabecular bone at the metaphysis in the femurs of both female and male Wnt4 cKO mice. Furthermore, femoral areal BMD and the cortical area were decreased in female Wnt4 cKO mice. Mild reductions were noted in the vertebral BMD and vertebral BV/TV of male Zbtb40 knockout mice. In addition, femur BMD in female and male KO mice and vertebral BMD in female KO mice were similar to those in control mice. The osteoporotic phenotype in Prrx1-Cre; Wnt4^*fl/fl*^ mice was milder than that in Prrx1-Cre; Wnt1^*fl/fl*^ mice even with the differences in the bone types and ages of these mice, suggesting that other Wnt ligands, including Wnt1, compensate for the function of Wnt4 in Wnt4 cKO mice.

### Wnt5a

A low bone mass with impaired bone formation and resorption has been reported in Wnt5a^+/–^ mice [9]. To investigate the roles of osteoblast-derived Wnt5a in bone formation and resorption, osterix-Cre; Wnt5a^fl/fl^ mice were generated, and their bone phenotypes were analyzed. Osterix-Cre; Wnt5a^fl/fl^ mice also showed a decreased bone mass with impaired bone formation and resorption. These findings indicate that Wnt5a from osteoblast-lineage cells promotes bone formation and resorption. In vitro studies using Wnt5a^–/–^ calvarial osteoblasts demonstrated that Wnt5a enhanced Wnt3a-induced Wnt/β-catenin signals and promoted osteoblast differentiation [29]. Cathepsin K is highly expressed in osteoclasts; therefore, recombination has been suggested to specifically occur in osteoclast-linage cells in Ctsk-Cre mice [30]. However, a recent lineage tracing study revealed that Ctsk-Cre-induced recombination occurred in periosteal stem and osteoclasts [31]. The bone phenotype of Ctsk-Cre; Wnt5a^fl/fl^ mice was investigated [32]. These mice exhibited decreases in BV and cortical bone thickness with impaired bone formation and normal bone resorption. These findings suggest that skeletal stem cells labeled by Ctsk-Cre-derived Wnt5a are involved in osteoblast differentiation and bone formation. Therefore, periosteal cells including skeletal stem cells isolated from the hind limbs of Ctsk-Cre; Wnt5a^fl/fl^ mice did not show obvious defects in the expression of osteoblast maker genes such as Runx2, Osterix, and Col1a1, in cultures, suggesting that skeletal stem cell-derived Wnt5a acts on other osteoblast precursors rather than skeletal stem cells themselves, which, in turn, promote bone formation. We cannot rule out a possibility that osteoclasts-derived Wnt5a also promote bone formation. Further studies are needed to elucidate the mechanisms by which Wnt5a promotes bone formation.

### Wnt10b

Wnt10b is mainly expressed in pre-adipocytes and stromal vascular cells, but not in adipocytes [33, 34]. The overexpression of Wnt10b in adipose tissue using FABP4-Wnt10b mice resulted in reduction in white adipose tissue and marked increases in trabecular bone mass [35]. Furthermore, Wnt10b^–/–^ mice showed reduced bone mass due to a decrease in bone formation (approximately -30%). These findings indicate that adipose tissue-derived Wnt10b promotes osteoblast differentiation and positively regulates bone formation.

### Wnt11

A de novo heterozygous loss-of-function mutation in Wnt11 (NM_004626.2:c.677_678dupp.Leu227Glyfs ∗ 22) was detected in a 4-year-old boy with low BMD and fractures by whole-exome sequencing [36]. Heterozygous mutant osteoblastic U2OS cells carrying the same mutation showed reduced Wnt11 mRNA and protein levels, as well as the down-regulated expression of osteoblast differentiation marker genes and a marked decrease in mineralization ability. In Wnt11 mutant cells, the expression of β-catenin and Lrp5, which were involved in the Wnt/β-catenin pathway, and Ror2, which plays a role in the non-canonical pathway, was decreased, suggesting impairments in both canonical and non-canonical pathways. In addition, the expression of Opg, a target gene of the Wnt/β-catenin pathway, was decreased in the mutant cells. The addition of rhWnt11 restored the expression of Opg and increased that of Aixn2, suggesting that Wnt11 mainly activates the canonical pathway. Further studies using Wnt11 knockout mice are needed to further clarify the roles of Wnt11 in bone metabolism.

### Wnt16

A missense SNP (Thr > Ile; rs2707466) located in the Wnt16 gene has been associated with cortical bone thickness [37]. This SNP, as well as the nonsynonymous SNP rs2908004 (Gly.Arg), showed a significant genome-wide association with forearm BMD. Female Wnt16^–/–^ mice had cortical bones that were 27% thinner at the femur mid shaft than those in their wild-type littermates as well as bone strength measures that were between 43 and 61% lower in both the femur and tibia [37]. The strongest forearm fracture signal at *WNT16* exhibited prominent bone-site specificity with no association with hip fractures [38]. To elucidate the mechanism by which Wnt16 regulates bone mass, the bone phenotypes of Wnt16^–/–^ mice were examined [39]. Wnt16 was highly expressed in osteoblasts on cortical bone surfaces. Wnt16^–/–^ mice were susceptible to spontaneous fractures due to decreases in cortical thickness. Consistent with the previous findings, BV/TV was normal in these mice. In Wnt16^–/–^ mice, the number of osteoclasts was increased in cortical bone, which, in turn, increased cortical porosity. Mechanistically, Wnt16 increased the expression of Opg through the activation of Wnt/β-catenin signals. Therefore, osteoblast-derived Wnt16 suppresses osteoclast differentiation to maintain cortical bone thickness. An in vitro study confirmed that Wnt16 suppresses osteoclast differentiation in Opg-dependent and independent manners [40].

### Others

To the best of our knowledge, there are currently no studies on the bone phenotypes of Wnt2, Wnt5b, Wnt7b, or Wnt10a mutants in humans and mice.

## Conclusions

This review introduced Wnt ligands in which mutations cause a low bone mass phenotype in humans and mice. Osteoblast lineage cells secrete various Wnt ligands that may act on surrounding cells locally, but not systematically. Many studies have examined Wnt1 mutations in humans and mice indicated that Wnt1-derived osteoblast lineage cells mainly regulate osteoblast differentiation and bone formation; however, Wnt1 expression levels are not very high. Future studies are needed to identify Wnt1-secreteing cells in bone tissues under physiological and pathological conditions, to clarify the mechanisms by which it acts on neighbor cells, and to develop molecules that specifically promote Wnt/β-catenin signals in bone.
